# Magnetic Flux Guides by Material Extrusion

**DOI:** 10.1002/advs.202517310

**Published:** 2025-12-19

**Authors:** Jorge Cañada, Steven W. Wright, Michail E. Kiziroglou, Eric M. Yeatman, Luis F. Velásquez‐García

**Affiliations:** ^1^ Microsystems Technology Laboratories Massachusetts Institute of Technology 77 Massachusetts Ave Cambridge MA 02139 USA; ^2^ Department of Electrical and Electronic Engineering, South Kensington Campus Imperial College London London SW72AZ UK

**Keywords:** 3D Printing, additive manufacturing, energy harvesting, magnetic flux concentrators, soft‐magnet

## Abstract

Additive fabrication of active materials with sub‐millimeter resolution can improve the performance and expand the functionality of sensing, actuation, and transduction in microsystems. In particular, the integration of soft magnetic materials of customized 3D geometries that guide and focus magnetic flux can increase inductive coupling and interaction forces. In this paper, a magnetic material extrusion method is used to fabricate flux concentration structures of arbitrary shapes. A magnetic permeability of 42 is experimentally demonstrated. Ring and H‐shape structures are used to evaluate their performance as inductive, power‐line energy harvesters. An output power density of 6.4 µW g^−1^ is demonstrated by open‐loop coupling to a 10 A, 500 Hz power line emulating an aircraft use case. The results are compared with similar ferrite and moulded material devices, which yield 17.3 and 2.4 µW g^−1^, respectively. In line with a simulation analysis, the experimental results show that materials with moderate magnetic permeability can provide competitive transduction performance, while offering unique customisation, accessibility, and design‐to‐prototype speed benefits. The proposed customisable magnetic flux‐concentration approach provides a simple, effective, and accessible method for enhancing the performance of magnetic and inductive sensing, actuating, and energy transduction devices.

## Graphical abstract

Magnetic flux guidance can increase inductive coupling and forces for magnetic sensors, actuators, and transducers. Here, a magnetic material extrusion method is used to fabricate flux concentration structures of arbitrary shapes with a magnetic permeability of 42. In line with simulation analysis, experimental results show that these materials provide competitive transduction performance while offering unique customization, accessibility, and design‐to‐prototype speed benefits.

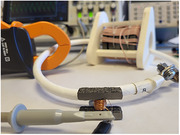

## 1 Introduction

Several technologies that are paramount to contemporary quality of life, such as electrical transformers, motors and generators, medical imaging systems, data storage, and vehicle/industrial automation systems, rely on induction, flux coupling, and magnetic force delivery. However, while these technologies perform well at the macroscopic scale, their underlying operating principles are challenging to scale down. This difficulty arises from the area‐dependent scaling of available flux, limitations in 3D coil structure fabrication by planar processes, and the incompatibility of high‐performance magnetic materials with standard microelectronic, or micro‐electro‐mechanical systems (MEMS) fabrication processes. These limitations hinder the exploitation of electromagnetic phenomena at the microscale for applications such as on‐chip sensing, energy harvesting, and actuation. As a result, other transduction mechanisms, such as piezoelectricity and electrostatic forces, which are more favorable in the MEMS scale, have been widely adopted in the state‐of‐the‐art of micro‐motors, micro‐generators, and miniature or chip‐integrated sensors and actuators. Nevertheless, recent progress in chip‐integration of magnetic materials, mainly based on deposition and sintering, shows promise for implementing devices of high performance and extended functionality, such as the piezoelectric AlScN devices integrated with NdFeB magnets reported in.^[^ ^1^ ^]^

To address the flux limitations in millimeter‐ and sub‐millimeter‐scale devices, the use of soft magnetic materials as flux guides has gained significant interest in recent years. In this approach, a soft magnetic structure is used to increase the magnetic flux density *B* at a location, both via magnetization and through geometrical funnelling: by guiding a given amount of flux from a larger cross‐sectional area to a smaller one, the magnetic flux density at the smaller section is increased. A miniature electromagnetic transducer (e.g., a coil, a Hall sensor, a magnetic actuator) situated at the position of a small cross‐sectional area benefits from the increased flux density, enhancing its performance. Various geometrical guiding or funnelling structures have been employed, including planar shields, U‐shape guides,^[^ ^2^ ^]^ trapezoidal tapers,^[^ ^3^, ^4^ ^]^ H‐structures,^[^ ^5^, ^6^, ^7^ ^]^ 3D structures like bent triangular panels ^[^ ^3^ ^]^ and bow‐tie structures,^[^ ^8^ ^]^ as well as rectangular or circular guides with a step reduction of cross‐sectional area,^[^ ^9^, ^10^ ^]^ among others. These flux funnelling structures have been shown to increase *B* at the transducer location, thereby enhancing the sensitivity of resonator magnetometers,^[^ ^11^ ^]^ magneto‐resistive sensors ^[^ ^12^, ^13^ ^],^ and current sensors.^[^ ^3^ ^]^ More recently, soft‐core structures with stepped‐down cross‐sectional area at the sensor location have been proposed to improve the sensitivity of superconducting magnetoresistive sensors.^[^ ^9^, ^10^ ^]^ Similarly, soft‐core rings with tapered cross‐sections have been proposed for high‐sensitivity fibre optic current sensors (FOCS).^[^ ^14^ ^]^ Integrated magnetic concentrators are also used to guide in‐plane flux to the perpendicular orientation in chip‐integrated sensors, enabling 3D field sensing by in‐plane fabricated Hall sensors.^[^ ^15^, ^16^ ^]^

In such sensing applications, the sensitivity improvement is proportional to the *B* increase achieved by the flux funnelling structure. Similarly, in conventional power devices such as transformers, actuators, generators, and motors, operating at higher *B* offers benefits including reduced device mass and volume, increased actuation force, stronger coupling, and lower coil resistance. The latter results in reduced losses and higher operating efficiency—for example, in inductive generators. Moreover, for applications where power maximisation is a key priority, such as energy harvesting or fast charging of portable electronics, the increased *B* provides a direct benefit on performance, as operating power scales with the square of *B*.^[^ ^5^, ^17^ ^]^ Therefore, flux funnelling is of particular importance for such applications. Indeed, a wide range of flux guides has been employed in energy harvesting devices. For example, H‐shaped,^[^ ^6^ ^]^ bow tie,^[^ ^8^ ^]^ and U‐shape flux flanking ^[^ ^2^ ^]^ structures have been employed in power line inductive energy harvesting, demonstrating performance increases of at least an order of magnitude. Notably, a prototype inductive energy harvester featuring a stepped cross‐sectional core, designed to harness structural currents running along railway tracks, delivered 40 mW at a 250 mm distance from a 200 A, 50 Hz emulated distributed structural current.^[^ ^18^ ^]^ Additionally, radial flux‐concentration bridges have been employed in pipeline infrastructure monitoring applications to increase the performance of magnetohydrodynamic energy harvesting.^[^ ^19^ ^]^

These advancements show great promise for the development of scalable, high‐performance magnetic and inductive sensors, actuators, and power transducers. However, their industrial adoption is subject to the availability of manufacturing technologies capable of producing custom‐shaped, 3D flux‐funnelling structures from materials with suitable magnetic properties. Notably, the need to adapt the core shapes to complex funnelling geometries and, potentially, to physically constrained environments (e.g., implantable biomedical devices), limits the practicality of using traditional magnetic materials such as ferrites, which are difficult to shape into nonstandard geometries.^[^ ^20^ ^]^ This limitation has motivated the exploration of alternative materials and fabrication techniques. In particular, additive manufacturing (AM) technologies, which stand out for their accessibility and suitability for small‐batch, customized production, have been used to fabricate arbitrary‐shape soft magnetic cores.^[^ ^21^ ^]^ Recently, a method capable of seamlessly fabricating custom magnetic structures of moderate relative permeability μ_*r*_, in the 30 range, was reported.^[^ ^22^, ^23^ ^]^ In parallel, it has been demonstrated that flux‐funnelling structures within the same permeability range can significantly enhance the performance of inductive energy harvesting devices.^[^ ^24^ ^]^

In this paper, the fabrication method introduced in ^[^ ^22^, ^23^ ^]^ is employed to fabricate magnetic flux concentrators of custom shapes designed for flux funnelling. The structures are integrated into inductive coil transducers, and their magnetic properties are characterised. The performance of the devices as power line energy harvesters is evaluated. For this purpose, an experimental setup corresponding to an aircraft sensing use case is employed.^[^ ^25^ ^]^ The results are compared against the performance of corresponding commercial moulded and ferrite material devices, also developed as part of this work. The potential of the proposed flux funnelling method for implementing high‐performance magnetic and inductive transducers is demonstrated and discussed. The objective is to demonstrate that materials with moderate permeability can provide inductive transduction performance comparable with the current state‐of‐the‐art, using flux guiding structures.

The flux funnelling concept is discussed in Section 2, including a simulation analysis of the effect of geometry and permeability on *B* concentration. The experimental method, including fabrication, assembly, and evaluation details, is presented in Section 3. The results are presented and discussed in Section 4, including magnetic and energy harvesting performance characterisation. Conclusions are drawn in Section 5.

## 2 Geometrical Magnetic Field Amplification Concept

**Figure 1:**
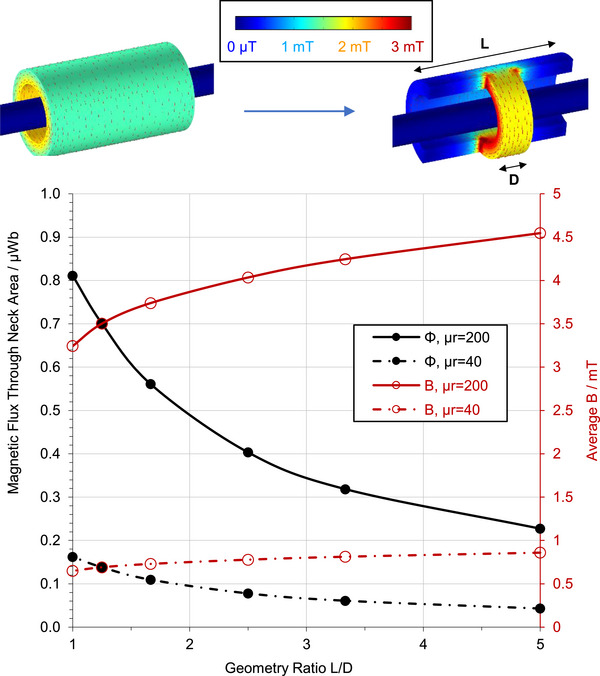
Geometrical magnetic flux‐guiding concept (top) and indicative finite element simulation (bottom) for a closed‐loop core. In the simulations, L was fixed to 50 mm, and D was varied between D = L and D = L/5. The conducting line is 10 mm in diameter and carries a 1 A RMS, 500 Hz current. The simulations were carried out using COMSOL Multiphysics. Note that the B displayed in the 3D rendering (top) corresponds to surface values, whereas the averages shown in the simulation graph (bottom) are higher as they include values from inside the material.

**Figure 2:**
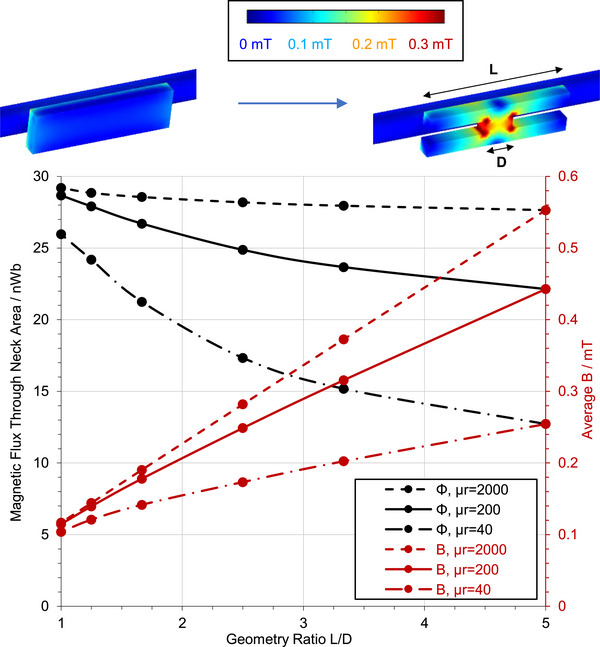
Geometrical magnetic flux‐guiding concept (top) and indicative finite element simulation (bottom) for an open geometry. In the simulations, L was fixed to 50 mm, and D was varied between D = L and D = L/5. The conducting line is 10 mm in diameter and carries a 1 A RMS, 500 Hz current. The simulations were carried out using COMSOL Multiphysics. As in Figure 1, the 3D B plots (top) illustrate surface values, whereas the averages in the simulation graph (bottom) include values from inside the material, resulting in higher values.

The electric field near a charged object can be significantly intensified at certain locations by employing sharp tip geometries, where high surface charge density results in strong electric field concentration. This phenomenon is commonly exploited in field emission devices such as electron beam sources. Analogously, sharp tips on permanent magnets or electromagnets lead to higher magnetic field concentrations and are used to achieve higher field strengths and field gradients in applications like magnetic force microscopy (MFM) and microscale magnetic resonance imaging (MRI).^[^ ^26^ ^]^ A simple way to illustrate the geometrical amplification of a magnetic field is through the concept of magnetic flux: a tapered magnetic material can guide a given flux through a smaller cross‐section, thereby increasing *B*. This phenomenon is illustrated in **Figure** **1** for a closed‐loop core case, and in **Figure** **2** for the open‐loop core case. For the finite element simulations presented in these figures, an overall air box of 0.7 m × 0.4 m × 0.4 m was defined, with magnetic insulation boundary conditions for the magnetic field. A Ø 10 mm cylindrical conductor along the 0.7 m direction of the air box was defined, with a 1 A root‐mean‐square (RMS), 500 Hz current source and sink at the boundaries.

Figure 1 presents the case of a closed‐loop soft magnetic core wrapped around a conducting line. If the cylindrical core structure is cut as shown by the arrow in Figure 1, top, the additional flux gathered by the longer semi‐cylinder (length *L*) is guided through the shorter semi‐cylinder (length *D*), leading to an increase of *B* in the short semi‐cylinder. The colour scale of *B* intensity and the *B* vector field direction (red arrows) shown in the Figure quantify this phenomenon for the case of a core with μ_*r*_ =  200 wrapped around a Ø 10 mm conducting line that carries a 1 A RMS, 500 Hz current. The simulation results manifest flux guiding, as *B* clearly intensifies in the short semi‐cylindrical section of the core. Figure 1, bottom, shows the simulated total flux and average *B* through the short semi‐cylinder cross‐section as a function of the *L*/*D* ratio. The data show that, for μ_*r*_ =  200 and *L*/*D*  =  5, *B* only increases by a factor of 1.40—significantly less than the geometrical ratio. This effect can be explained as follows. The flux lines flowing through the lateral semi‐cylindrical parts of the soft core close around the current line through longer paths, as they must deviate such that they pass through the short length bridge. Such longer paths impose higher reluctance, which translates to lower flux and, in turn, weaker magnetization of the lateral semi‐cylindrical sections. Hence, the contribution of the lateral core sections to the *B* across the bridge drops as distance to the bridge increases. In summary, in closed‐loop magnetic cores, the core is already strongly magnetised at the bridge location, and the lateral flux‐funnelling material provides only a small additional flux, ≈40% for μ_*r*_ =  200 and 32% for μ_*r*_ =  40 for a geometrical ratio of 5. Although modest, this increase may still be significant for sensing and, especially, for energy harvesting applications, in which power output scales with *B* ^2^.

The case of an open‐loop magnetic core situated next to a conducting line is illustrated in Figure 2. In this scenario, the core magnetisation is a fraction of that corresponding to its nominal permeability μ_*r*_ due to the presence of air in the closed flux path, which presents a high reluctance to the flux flow. If the core is cut to an H‐shape, as shown by the arrow in Figure 2, top, with flange length *L* and bridge length *D*, the flux flowing through the flanges is guided to the shorter length bridge, leading again to an increased *B* at the bridge. In this case, however, the gain is much larger than in the closed‐loop scenario. Since the total reluctance of the flux path around the conducting line is dominated by the high reluctance of air, the relative reluctance increase introduced by the longer path along the flanges is very small. Thus, the *B* increase is not limited by the longer paths needed to be travelled to pass through the bridge, leading to a substantially larger *B* gain. As before, the effect is quantitatively studied by simulation for a 1 A RMS, 500 Hz current. The total flux and average *B* through the bridge is plotted as a function of *L*/*D* in Figure 2, bottom. A *B* increase by a factor of 3.9 is demonstrated for μ_*r*_ =  200, at *L*/*D*  =  5. For μ_*r*_ =  2000 and μ_*r*_ =  40, the corresponding gains are 4.7 and 2.4 respectively. The results demonstrate that, for high permeability materials, the flux funnelling ratio can indeed approach the geometrical cross‐section scaling ratio, in open‐loop fields. In addition, even for moderate and low permeabilities, flux funnelling can provide a very high *B* enhancement. It is worth noting that materials of very high permeability, such as high‐performance ferrites, are difficult to machine into custom shapes. Therefore, the benefit of their use is limited in practice to the contribution of μ_*r*_ alone. Meanwhile, materials of moderate permeability can benefit from the flux enhancement effects of both their permeability and geometrical guiding. The simulation results presented in Figure 2 reveal that the benefit of increasing μ_*r*_ alone, without geometrical flux guidance, is negligible. For example, increasing the permeability from μ_*r*_ =  40 to μ_*r*_ =  2000 at *L*/*D*  =  1 only increases flux density from 0.104 to 0.115 mT. Meanwhile, geometrical flux guiding through smaller cross sections, even for materials of moderate permeability, leads to a large *B* increase, indicatively 390% at μ_*r*_ =  200 and 240% at μ_*r*_ =  40, for a geometrical ratio *L*/*D*  =  5. These observations suggest that, while increasing μ_*r*_ is beneficial, performance is primarily determined by geometrical funnelling rather that material permeability and, therefore, materials of moderate μ_*r*_ of customisable shapes may be preferable over materials of high μ_*r*_ that are difficult to process (e.g., ferrites), for increasing magnetic coupling for small scale sensing, actuating, and power transduction devices. This observation is experimentally studied and discussed in the rest of this paper.

## 3 Experimental Section

### 3.1 Fabrication of Freeform Soft Magnetic Cores

The simulations reveal that the ability of magnetic cores to concentrate magnetic flux in the vicinity of a power line is determined by their magnetic permeability and their geometry, with μ_*r*_ being the dominant factor in the case of closed‐loop cores, while geometry dominates the performance of open‐loop cores. This finding motivates the choice of a manufacturing technology that enables the fabrication of high‐permeability cores with arbitrary shapes. Moreover, in many power line energy harvesting applications, the morphology of the harvesting device needs to fit an already existing infrastructure, imposing constraints on the shape of the magnetic core, and highlighting the need for a manufacturing technology capable of producing cores with custom, intricate shapes.

**Figure 3:**
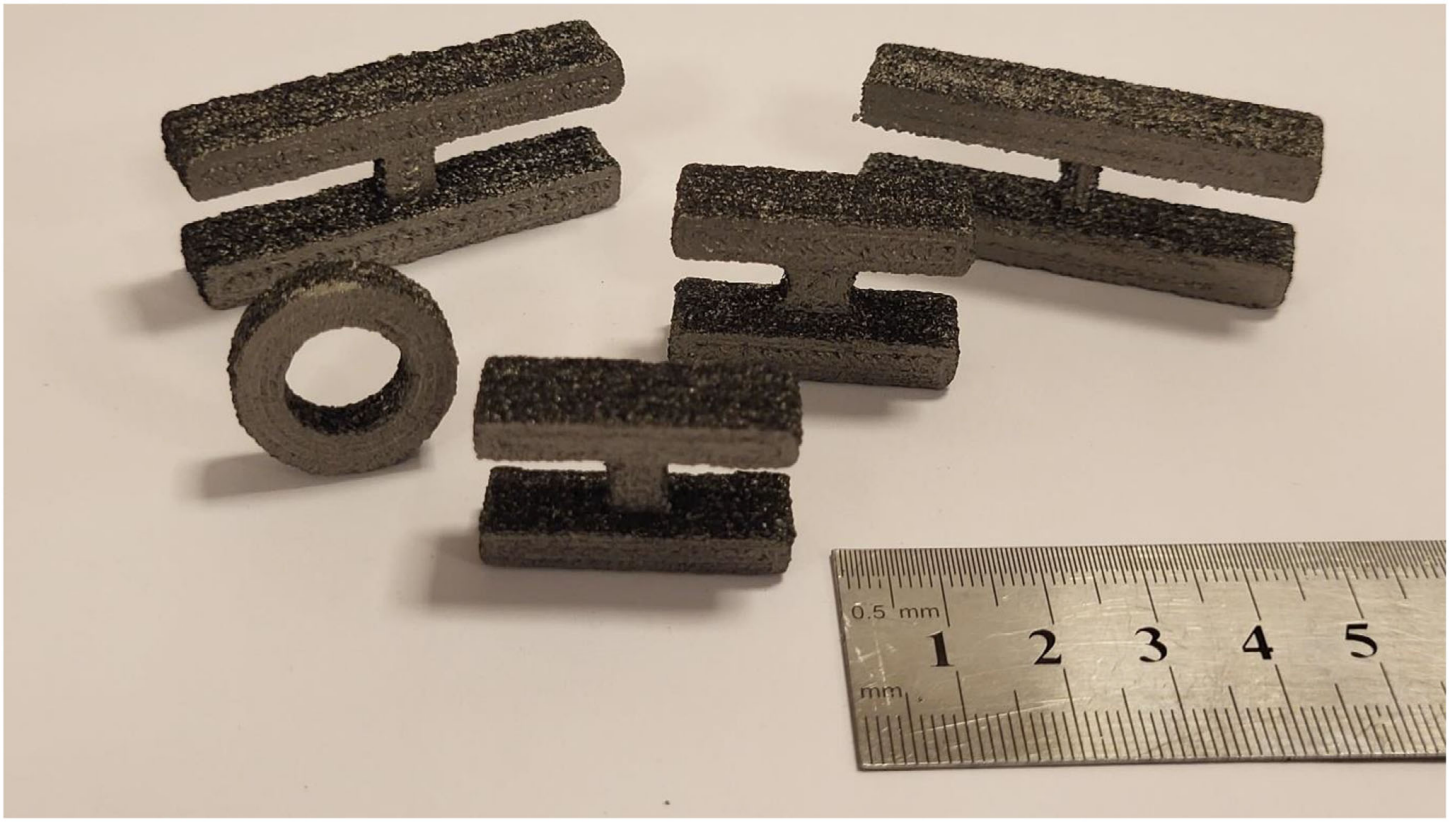
Magnetic guides fabricated via material extrusion.

While traditional manufacturing technologies are effective for the high‐volume production of cores with standardized geometries, they are not suited for manufacturing custom‐shaped cores. Ferrite core fabrication in particular incurs elevated tooling and setup charges (in the order of several $1000), and development times of many weeks, when considering the production of a new geometry. Meanwhile, AM technologies, characterized by their ability to produce design‐accurate, custom‐shaped parts, are well‐suited for the fabrication of cores of arbitrary shapes. Additionally, AM technologies are typically low‐waste and cost‐efficient. In this work, soft magnetic cores with various geometries were 3D‐printed via material extrusion (**Figure** **3**) and characterized to evaluate their performance in power line energy harvesting applications.

**Figure 4:**
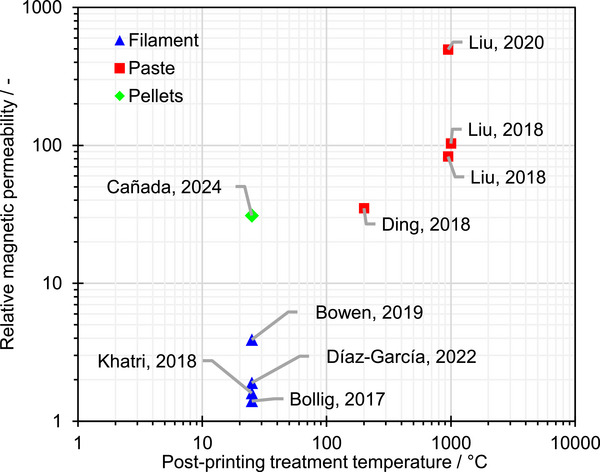
Comparison of relative magnetic permeabilities of 3D‐printed soft magnetic components reported in the literature. Devices that do not require heat postprocessing were assigned a treatment temperature of 25 °C (i.e., ambient temperature).

Material extrusion is an AM technology that creates parts by pushing feedstock through a nozzle and depositing it layer by layer. It is one of the most widely accessible AM technologies, and it is suitable for the fabrication of magnetic components.^[^ ^21^ ^]^ Recent studies have reported the use of material extrusion‐compatible, soft magnetic feedstock in the form of filaments ^[^ ^27^, ^28^, ^29^, ^30^, ^31^, ^32^, ^33^ ^]^, pastes^[^ ^31^, ^32^, ^33^ ^]^ and pellets ^[^ ^22^, ^23^ ^]^ (**Figure** **4**). Thermoplastic‐based filaments are the standard feedstock for material extrusion, as they enable tight control of material flow, and they can be embedded with particles of functional materials to impart properties such as electrical conductivity and magnetism. However, producing 3D printing‐compatible filaments with high filler content is challenging, limiting the functional performance of filament‐printed parts.^[^ ^34^ ^]^ Consequently, the relative magnetic permeability of the filament‐based soft magnetic cores reported in the literature is low (below 10). Meanwhile, soft magnetic devices printed from pastes have been shown to reach magnetic permeabilities of several hundred. However, pastes require sintering at 950 °C or higher temperatures after printing, and their rheology introduces challenges for achieving dimensional accuracy. In contrast, pelletized feedstock can reportedly attain moderate permeabilities of ≈30 while not requiring post‐processing treatments. Therefore, soft magnetic pellets enable a compromise between magnetic permeability and manufacturability that aligns with the goal of this study—to demonstrate the ability of materials with moderate magnetic permeability to enhance the performance of induction transducers via custom‐shape flux guiding structures.

**Figure 5:**
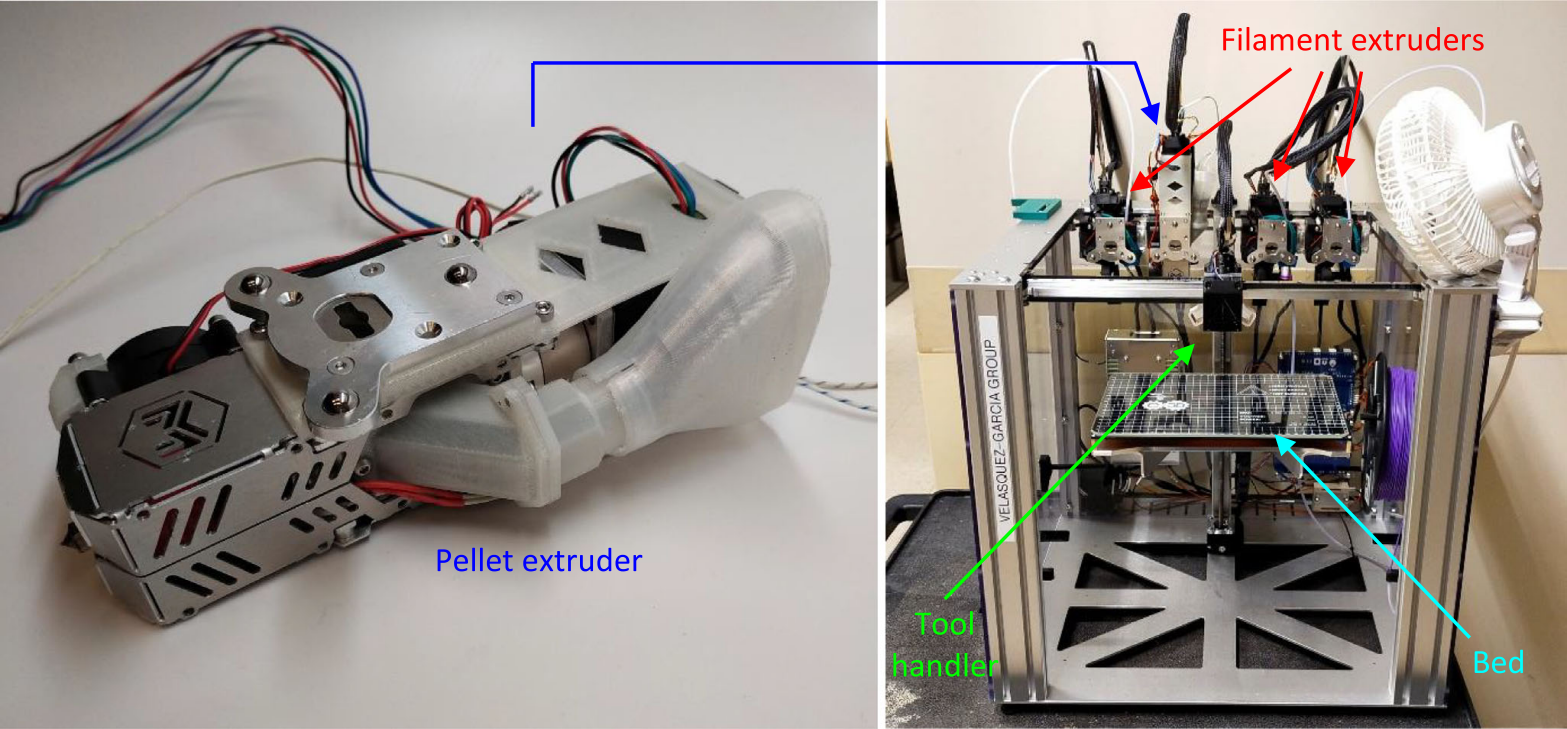
Customized Mahor v4 pellet extruder (left) and E3D ToolChanger 3D printer featuring three filament extruders and one pellet extruder (right).

**Figure 6:**
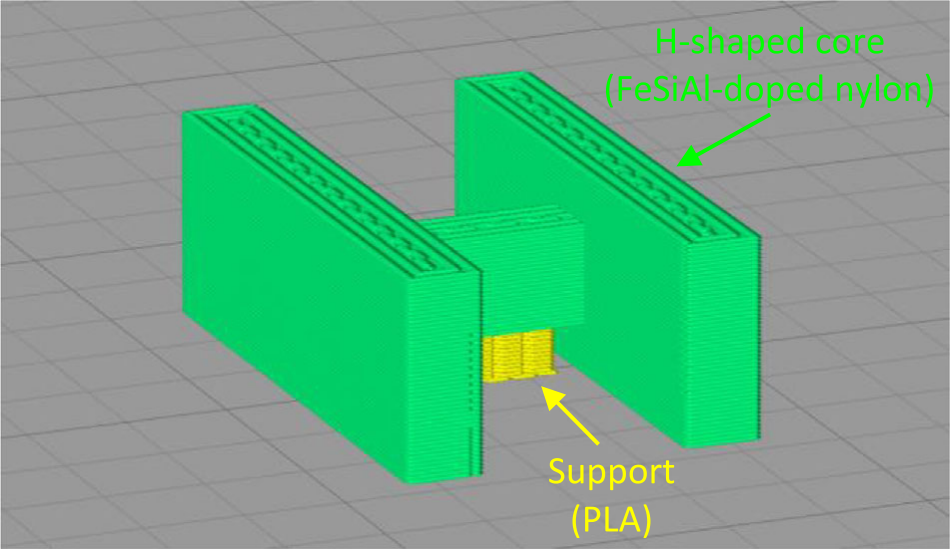
Rendering of H‐shaped core and bridge support structure in the slicer software Simplify3D (Simplify3D, Cincinnati, OH, USA).

In this work, pellets of nylon 12 doped with FeSiAl particles at 86% in weight (MATE Co., Okayama, Japan)—the same material explored in ^[^ ^22^, ^23^ ^]^—were used to fabricate soft magnetic cores via pellet extrusion. To do so, a Mahor v4 Pellet Extruder (Mahor.xyz, Spain) was mounted on a customized E3D Motion System and ToolChanger 3D printer (E3D‐Online, Chalgrove, Oxfordshire, United Kingdom) (**Figure** **5**). The E3D ToolChanger system can accommodate up to four independent tools, which can be automatically picked up and used sequentially during a print job, enabling the monolithic fabrication of multi‐material devices. Furthermore, the open‐source nature of the system and its customization‐friendly design enable the integration of personalized tools, such as the pellet extruder used in this work. The 3D printing system, including the pellet extruder, has a total cost under $4000, and it can fabricate a wide range of geometries—with a minimum feature size of ∼0.8 mm and a maximum build volume of 300 mm × 200 mm × 250 mm—without requiring setup changes. For core fabrication, only two out of the four toolheads were used: one to extrude the soft magnetic material and another to print support structures in polylactic acid (PLA), which held the bridges of the H‐shaped cores during printing, and were later removed (**Figure** **6**). Table 1 summarizes the key printing parameters used in the fabrication process. The dimensional accuracy of the pellet extrusion process was sensitive to the material and printing parameters used. In this work, the extrusion width parameter was set to a value smaller than the diameter of the nozzle, resulting in overlapping nozzle sweeps and overextrusion. This choice of printing settings facilitates the production of fully dense parts, but can also result in oversized dimensions in the plane parallel to the printing substrate—the horizontal plane—due to horizontal overflow of excess material. In this work, the effect of overextrusion was countered by shrinking the horizontal dimensions of the part designs by 1 mm—determined experimentally—to make the final dimensions of the 3D‐printed components match the nominal dimensions. The soft magnetic material used contains metal flake powder of up to 150 µm in length. Such a large particle size resulted in rough lateral surfaces, but it was not found to significantly affect the rheology of the material or the dimensions of the printed parts. No significant dimensional variation across samples was detected.

**Table 1:** Key 3D printing parameters used to fabricate the soft magnetic cores, as defined in the slicer software Simplify3D.

	Core	Support
Nozzle diameter (mm)	0.8	0.4
Extrusion multiplier (‐)	1.00	1.00
Extrusion width (mm)	0.75	0.48
Layer height (mm)	0.2	0.2
Extrusion temperature (°C)	270	200
Bed temperature (°C)	90	90
Speed (mm/min)	1000	1000

Employing this method, rings with 13 mm inner diameter, 21 mm outer diameter, and 6.35 mm length were fabricated (bottom‐left of Figure 3). The dimensions were selected to match commercial ferrite rings that were purchased for comparison. In addition, H‐shaped flux funnelling devices of various sizes were fabricated. For the experiments of this paper, the H‐shaped devices with square bridge cross‐sections were used (bottom and top left in Figure 3). Each device was fabricated in under one hour, and had a cost of under $1 in materials.

### 3.2 Transducer Fabrication

**Figure 7:**
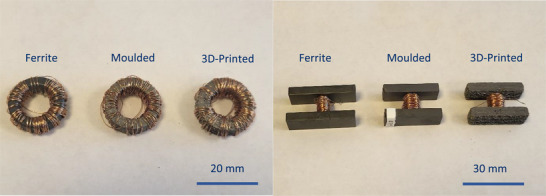
Closed‐loop ring (left) and open, H‐shaped (right) core‐and‐coil transducers.

Inductive toroidal transducers were fabricated using the 3D‐printed soft core rings. Additionally, for comparison, equivalent transducers were constructed using commercial ferrites and custom‐moulded rings of identical geometry. The custom moulded material was provided by Max Baermann GmbH. A 100‐turn coil was wound around each ring using enamelled Ø = 0.2 mm Cu wire (**Figure** **7** left). The ring transducers were used in this work primarily to characterise the magnetic properties of the materials. While their output voltage is analysed in Section 4.2 and their potential as energy harvesting devices is discussed, they do not provide geometrical guidance, and therefore, an evaluation of their output power was beyond the scope of this paper. Closed‐loop flux‐guide energy harvesting implementations (scenario depicted in Figure 1) have been proposed and analysed in.^[^ ^25^, ^35^ ^]^

Similarly, inductive H‐shaped transducers were prepared using H‐shaped cores to evaluate the flux funnelling performance of the materials in open‐loop configurations (scenario depicted in Figure 2. H‐shape cores with length *L*  =  30 *mm*, a 5 mm × 5 mm rectangular bridge cross‐section, 20 mm total height, and 10 mm width were used. A 100‐turn coil was wound around the bridge section of each H‐shaped core, using the same Ø 0.2 mm enamelled Cu wire (Figure 7 right). The H‐shaped transducers were evaluated as open‐loop inductive energy harvesters for power lines using the experimental setup described in the following section.

### 3.3 Power Line Energy Harvesting Evaluation Setup

**Figure 8:**
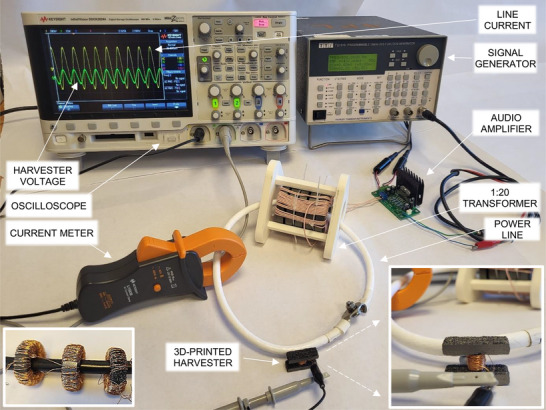
Photograph of the power‐line harvesting evaluation setup. Left inset: Installed ring transducers. Right inset: Close‐up of the H‐shaped 3D‐printed transducer.

The experimental setup used for material and device characterization (Figure 8) emulates a power line with cable diameter Ø ≈10 mm, carrying currents of up to 20 A RMS, in the 50 Hz–1 kHz frequency range. It was based on a commercial audio amplifier coupled with a custom, 1:20 current transformer with ferrite core rings, used to obtain the required current and frequency range on the power line cable. The input of the amplifier was driven by a TTi TG1010 programmable function generator. The secondary coil of the current transformer was the power line cable, connected in a single loop. The maximum line current was limited by the resistance of the cable loop. Higher current values can be obtained by using a cable of higher conductive cross‐section and a low‐resistance contact method between the cable ends, such as high contact area clamps. The power line current was monitored using a Fluke 787B process meter and a Keysight U1583 clamp probe. A 4‐channel Keysight DSOX 2024A oscilloscope was used to monitor the audio amplifier waveform and the output of the transducers under test in open‐circuit conditions, as well as in connection with resistive loads consisting of a resistance box. This apparatus can be used for characterizing the magnetic properties of soft‐core materials using ring inductive transducers, such as those described in the previous section, as well as for evaluating inductive energy harvesting devices for power line applications.

To characterize the magnetic core materials, the spatial average magnetic flux density *B* in the material can be measured as a function of spatial average magnetic field *H* using a ring core (Figure 8, left inset). With the power line cable going through the centre of the ring core, the average magnetic field inside the core can be calculated from the power line current *I*, using Ampere's law around the middle ring perimeter:

(1) $$I=\oint_{c}Hdl\Rightarrow H=\frac{I}{c}$$

At the same time, the magnetic flux density can be calculated from the open‐circuit coil voltage *V* through Faraday's law:

(2) $$V=-N\frac{\partial \Phi }{\partial t}\Rightarrow B=-\frac{1}{NA}\int_{0}^{t}Vdt$$

where Φ, *N*, and *A* are the flux, the number of coil turns, and the ring cross‐section area, respectively. Thereby, by monitoring and recording *I* and *V* for a few periods of an alternating current (AC) power line current, the *B* − *H* curve of the material can be obtained. As a complementary characterisation method, a Wayne Kerr 6500B spectroscopic impedance analyser was used to measure the impedance of the transducers as a function of frequency, in order to extract their resistance, inductance, and average core relative permeability.

The evaluation of ring and H‐shaped transducers as power line energy harvesting devices can be performed by measuring the transducer output voltage in open‐circuit conditions, as well as under different loads, in order to identify the output impedance and the maximum power point of the transducers. The loads can be purely resistive, emulating a power‐consuming device such as a sensor node, or complex impedances to include reactance compensation circuitry.^[^ ^25^ ^]^ Given that the focus of this work was on material performance, transducers with only a small number of coil turns were tested, and reactance compensation was not contemplated in the evaluation of energy harvesting output power. For use‐case implementations, coils with higher numbers of turns and reactance cancellation could be developed and tested using the same evaluation setup.

## 4 Results and Discussion

### 4.1 Magnetic Permeability Measurements

**Figure 9:**
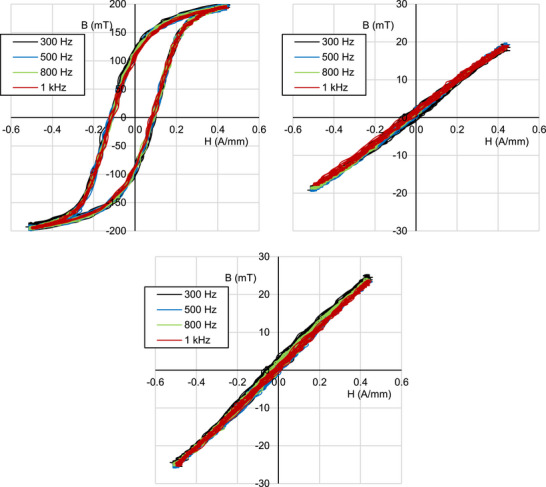
Measured B‐H characteristics of commercial ferrite (top‐left), moulded (top‐right), and 3D‐printed (bottom) rings.

**Figure 10:**
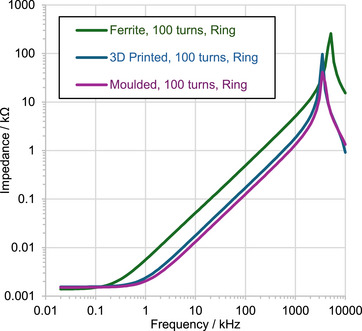
Impedance measurements of commercial ferrite, moulded, and 3D‐printed rings.

Using the evaluation setup and method described in Section 3, the *B* − *H* characteristic curves of the ferrite, moulded, and 3D‐printed materials were obtained for different frequencies in the 300 Hz to 1 kHz range (**Figure** **9**). This frequency range is of particular interest for aircraft power line sensors and applications.^[^ ^36^ ^]^ The commercial ferrite material exhibits a typical hysteresis curve, with an average μ_*r*_, measured by linear fitting in the region between − 95% and 95% of maximum *B*, of 450 at 300 Hz. The μ_*r*_ for 500 Hz, 800 Hz and 1 kHz was found to be 470, 490 and 520, respectively. A remanence of around *B_r_* =  0.12 *T* is also observed. On the other hand, the moulded and the 3D‐printed materials exhibit lower magnetization, with μ_*r*_ of ≈32 and 42, respectively, throughout the frequency range, and without noticeable hysteresis and remanent magnetization in the range of *H* studied. These measurements demonstrate a frequency independence of the magnetic properties of the materials at this low frequency band. The results are summarized in Table **2**, which also includes μ_*r*_ values calculated from spectroscopic impedance analysis measurements in the 1 kHz to 1 MHz frequency range (**Figure** **10**) for comparison. The measured relative permeabilities of the moulded and 3D‐printed materials using either experimental method were similar. In contrast, the impedance‐based permeability for the ferrite material is significantly lower than that obtained from its *B* − *H* performance. The cause of this discrepancy has not yet been identified. On the other hand, the linearity of the measured impedance as a function of frequency in the double logarithmic plots of Figure 10 for all materials between 1 Hz and 1 kHz shows a constant inductance and permeability at this frequency range, in agreement with the weak frequency dependence of the *B* − *H* curves of Figure 9. Beyond 1 kHz, a resonance behaviour is observed in Figure 10. This resonance is specific to the coil used in the transducers, and therefore, no conclusions are drawn about the properties of the materials in this frequency range.

**Table 2:** Measured magnetic permeability of commercial ferrite, moulded and 3D‐printed, rings.

Method	B–H curves				Impedance
f / Hz	300	500	800	1000	10^3^ – 10^6^
Ferrite	450	470	480	520	128
Moulded	32	33	32	32	31
3D‐Printed	42	42	42	41	44

While the permeability of the 3D‐printed material used in this paper is an order of magnitude lower than that of a commercial ferrite, it is higher than that of the custom moulded material by 30%. Also, according to the analysis presented in Section 2, this moderate permeability may be adequate for effective flux funnelling, especially in an open magnetic loop configuration. This is experimentally investigated in Section 4.3.

### 4.2 Closed Magnetic Loop Performance

**Figure 11:**
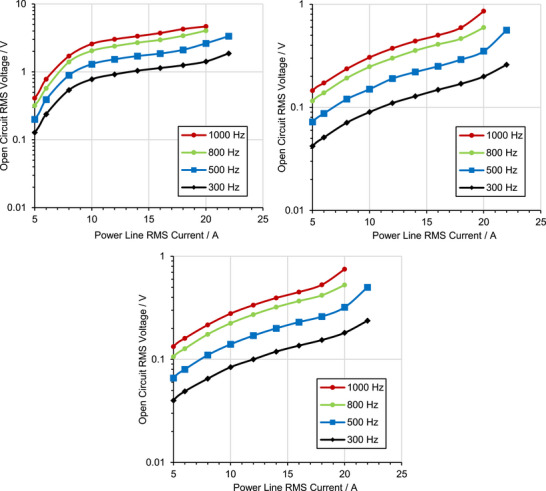
Open‐circuit RMS voltage of commercial ferrite (top‐left), moulded (top‐right), and 3D‐printed (bottom) magnetic ring transducers, from a 10 A RMS current of various frequencies.

The potential of using the proposed 3D printing technique to implement closed magnetic loop transducers was investigated by measuring the open‐circuit RMS voltage induced across the coils wound around ring cores, for different power line currents at different frequencies. The results are shown in **Figure** **11** for the ferrite, the moulded, and the 3D‐printed material cases. As expected for closed‐loop cores, the results show significant inductive coupling for all three cases despite the small number of coil turns used. Indicatively, for a 10 A RMS, 500 Hz power line current, the RMS voltage across the transducer coils is 1.3 V, 0.15 V, and 0.14 V for the ferrite, moulded, and 3D‐printed materials, respectively. The ferrite provides one order of magnitude higher voltage, corresponding to its similarly higher. μ_*r*_ measured and reported in Section 4.1. The voltage dependence on the line current amplitude follows a linear pattern (note the semilogarithmic scale of Figure 11) up to 10 A for the ferrite material, and it gradually saturates thereafter due to magnetisation saturation. For the moulded and 3D‐printed material devices, transducer voltage increases linearly with line current up to 20 A. The non‐linearities observed beyond 20 A for all three devices are due to signal distortion originating at the custom transformer of the evaluation setup. The frequency dependence also follows a linear pattern in accordance with Faraday's law of induction.

The induced voltage level obtained from the ferrite material is adequate for implementing energy harvesting power supplies suitable for autonomous low‐power electronic systems such as wireless sensor nodes. The lower voltage obtained by the moulded and 3D‐printed materials can be scaled up by increasing the number of coil turns. Indicatively, a tenfold increase in turns would provide adequate coupling while maintaining a practical overall device size and cost. Further transducer power density increase by using flux funnelling structures is possible,^[^ ^25^ ^]^ though limited for closed‐loop cores as demonstrated in Section 2. In conclusion, the results of Figures 9 and 10 show that the proposed 3D‐printed material has a moderate closed‐loop energy harvesting performance, and that higher μ_*r*_ is desirable, as coupling is dominated by the μ_*r*_ scale, in line with the simulations of Section 2.

### 4.3 Open Magnetic Loop Performance

**Figure 12:**
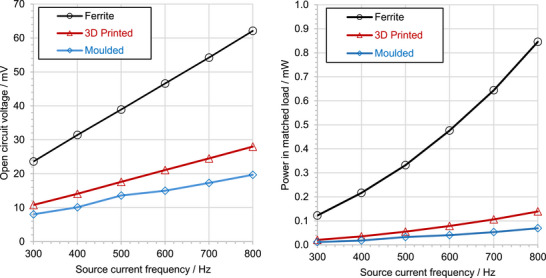
Open‐circuit RMS voltage (left) and corresponding matched‐load power output (right) of commercial ferrite, moulded, and 3D‐printed H‐shaped core‐and‐coil transducers, from a 10 A RMS power line current of various frequencies.

The open‐loop flux funnelling performance of the H‐shaped ferrite, moulded and 3D‐printed transducers was evaluated using the experimental setup described in Section 3.3. The transducers were installed on the side of the power line, with the H‐shape flanges parallel to the power line cable cylinder axis, and the bridge tangential to the cable, at a distance of 5 mm from the cable surface. This orientation corresponds to the simulated geometry shown in Figure 2, top right. The open‐circuit voltage of each transducer was measured for various line currents and frequencies. Indicative measurements for a 10 A RMS line current are shown in **Figure** **12**, left, as a function of frequency. A linear frequency dependence is observed as expected. The ferrite transducer provides a considerably higher voltage than the moulded and the 3D‐printed materials, due to its larger μ_*r*_. The 3D‐printed material provides a somewhat larger voltage than the moulded one, also in line with the corresponding measurements. μ_*r*_ values. The ferrite to 3D‐printed voltage ratio is 2 throughout the frequency range, which is much smaller than the permeability ratio of 10. This result is in line with the simulated *B* comparison of Figure 2, bottom, which indicates a similarly weaker dependence of voltage on μ_*r*_ for open magnetic loop devices. This smaller μ_*r*_ dependence can allow materials of moderate μ_*r*_ to be effectively used as flux funnels in open loop devices.

**Figure 13:**
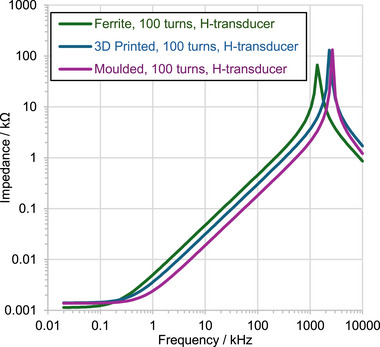
Impedance measurements of commercial ferrite, moulded, and 3D‐printed H‐shaped core‐and‐coil transducers.

To evaluate the maximum output power capabilities of the transducers, the impedance of the devices was characterized using an impedance analyzer (**Figure** **13**). The direct current (DC) coil resistances were measured at 1.14, 1.40, and 1.41 Ω for the ferrite, moulded, and 3D‐printed material devices, respectively. The corresponding inductances were measured at 0.78, 0.29, and 0.49 mH in the 1 kHz–0.5 MHz range. The maximum output power occurs at impedance matching conditions, at which the load impedance is matched. *Z_L_* is equal to the complex conjugate of the transducer output impedance *Z_OUT_*, i.e., when $Z_{L}=Z_{OUT}^{*}\;$. This can be achieved by a maximum power point tracking system including reactance compensation capacitors.^[^ ^25^ ^]^ The corresponding maximum power is shown in Figure 12, right. In contrast with the closed‐loop case presented in Section 4.2, which implies a power ratio of 100 between the ferrite and the 3D‐printed material, the ratio in the open‐loop case is less than 10. Indicatively, for a 10 A RMS, 500 Hz line current, powers of 332, 33, and 55 µW are demonstrated for the ferrite, moulded, and 3D‐printed materials, respectively.

A summary of device features and performance parameters is presented in Table **3**, including mass measurements for each device and the performance of a coreless coil.^[^ ^24^ ^]^ The power per total transducer unit mass, *P_m_* =  *P*/*m*, is found to be 17.3, 2.4, 6.4, and 1.2 µW g^−1^ for the ferrite, moulded, 3D‐printed, and coreless transducers, respectively. Using the coreless coil as reference, the power and power‐per‐unit‐mass gain factors are calculated as η  =  *P*/*P*_0_ and η_*m*_ = *P_m_* /*P*_*m*,0_, where *P*_0_ and *P*_*m*,0_ are the coreless coil power and power per unit mass, respectively. Notably, the power density of the 3D‐printed transducer is more than a third of that obtained by the commercial ferrite device. The experimentally measured performance of a 3D printed prototype of the same geometry but with longer flanges (*L*  =  50 *mm*, top‐left in Figure 3) is also included in Table 3. A power output of 71.4 µW, 30% larger than the 30 mm‐long printed prototype, was measured. While this increase is lower than the 66% geometrical ratio increase, it demonstrates that the μ_*r*_ of the printed material is adequate for providing higher flux concentration, and hence output power, by geometrical ratio scaling, as predicted by the simulation results in Figure 2.

**Table 3:** Energy harvesting performance of the H‐shaped magnetic flux concentrator transducers.

Quantity	Mass	µ_r_	L	R	P	η	P/m	η/m_r_
Unit	g	‐	mH	Ω	µW	‐	µW/g	‐
Ferrite	19.2	470	0.78	1.14	332	63.8	17.3	3.0
Moulded	13.6	33	0.29	1.4	33	6.3	2.4	0.4
3D‐printed	8.6	42	0.49	1.41	55	10.6	6.4	1.1
3D‐printed, long	13.7	42	0.49	1.5	71.4	13.7	5.2	0.9
Coreless	0.9	1	0.035	1.33	5.2	1.0	5.8	1.0

Note: L: Inductance at 500 Hz, R: DC coil resistance, P: Power from 10 A RMS, 500 Hz current, η: Power gain factor, P/m: Power per unit mass, η/m_r_: Power‐per‐unit‐mass gain factor with respect to the coreless coil (m_r_ is the mass ratio).

The demonstrated output power can be scaled up by increasing the number of coil turns. For example, as in the closed‐loop case, a tenfold increase in turns can be implemented without a significant size and mass overhead. Moreover, the overall core‐and‐coil transducer can be designed to fit the power, voltage, and size specifications of a given application. In energy harvesting transducers, the ratio of core and coil mass can be optimised for maximum power density, as shown in.^[^ ^5^ ^]^

The results demonstrate that both material permeability and core geometry play significant roles in magnetic field amplification, offering performance enhancements for closed‐ and open‐loop energy harvesting applications. Therefore, the ability to fabricate high‐permeability cores with custom shapes is desirable for the implementation of high‐performance power line energy harvesters.

The 3D‐printed cores fabricated in this work leverage the ability of AM to fabricate custom‐shaped, magnetically functional components. However, they exhibit modest permeability, which limits their energy harvesting performance. Recent studies on 3D‐printed magnetics have shown that cores with higher magnetic permeability can be fabricated by combining material extrusion AM with sintering (i.e., thermal postprocessing at temperatures near 1000 °C).^[^ ^32^, ^33^, ^37^ ^]^ While sintering typically introduces additional cost, extended manufacturing times, and potential geometric distortion, the ability to produce cores with significantly higher magnetic permeability (up to one order of magnitude greater) while maintaining shape flexibility makes this approach attractive for the fabrication of high‐performance power line energy harvesters.

The soft magnetic material used in this study (FeSiAl‐doped nylon) contains metal flake powder of up to 150 µm in length. The presence of such large‐sized metallic particles results in the need to use a wide nozzle to avoid clogging, imposing a large minimum feature size in the plane parallel to the printing substrate (≈0.8 mm in this work). Additionally, such a large particle size also constrains the out‐of‐plane minimum layer height that can be implemented (0.2 mm in this work). However, it is expected that the effect of flux guiding on transducer performance is more significant as the size of the transducer is reduced, due to the scaling of the magnetic flux with area. Therefore, it is of great interest to explore the synthesis of material extrusion‐compatible composite feedstocks incorporating smaller functional particles to enable the fabrication of millimetre‐ and sub‐millimetre‐scale magnetic flux guides.

## 5 Conclusion

The effectiveness of using 3D‐printed materials of moderate μ_*r*_ as flux guides for increasing the performance of magnetic transducers was studied in this paper. Finite element simulations show that, while in closed magnetic loop devices flux funneling is limited and performance is largely dependent on μ_*r*_, in open‐loop devices μ_*r*_ is less important, and flux funnelling can provide a *B* increase by a factor similar to the geometrical ratio of cross‐section scaling, even with materials of moderate μ_*r*_. The effect was studied experimentally using a 3D‐printed magnetic material suitable for implementing custom geometrical shapes. The material performance was characterised in comparison with a commercial ferrite and a custom moulded material, for closed‐loop and open‐loop device geometries. The material relative permeabilities were found to be between 450 and 520 for the ferrite, between 32 and 33 for the moulded, and between 41 and 42 for the printed material, at frequencies up to 1 kHz. In closed‐loop ring transducer devices, the ferrite transducer outperformed the moulded and 3D‐printed devices by an order of magnitude in terms of induced open circuit RMS voltage, confirming the dominant effect of μ_*r*_. In open‐loop, H‐shaped flux funnelling transducers, a weaker dependence on μ_*r*_ allowed the demonstration of significant relative performance of the 3D‐printed and the moulded materials. Indicatively, in open‐loop inductive energy harvesting operation, a power output of 332, 33, and 55 µW, from a 10 A RMS, 500 Hz power line current was demonstrated for the ferrite, moulded, and 3D‐printed materials, respectively. The corresponding power densities (per unit mass) are 17.3, 2.4, and 6.4 µW g^−1^. The ferrite to 3D‐printed material output power ratio is 6, significantly less than the 100 fold power ratio that is expected for a tenfold μ_*r*_ difference in closed‐loop devices. This result demonstrates that materials with moderate μ_*r*_ can be employed for fabricating inductive harvesters with customisable shapes to fit the requirements of sensing, actuating, and other applications involving magnetic flux. This is especially important for on‐chip transducers and applications requiring bespoke 3D shapes, in which high‐performance magnetic materials are challenging to integrate.

## Conflict of Interest

The authors declare no conflict of interest.

## Data Availability

The data that support the findings of this study are available from the corresponding author upon reasonable request.

## References

[1] T. Dankwort , M. Ahmed , S. Grünzig , A. Khare , B. Gojdka , in 2023 IEEE Int. Conf. on Mechatronics (ICM) , Loughborough, United Kingdom 2023, pp. 1–4.

[2] R. M. White , D. S. Nguyen , Z. Wu , P. K. Wright , Sensors (Basel) 2018, 18, 114. 29301354 10.3390/s18010114 PMC5795816

[3] K. Zhu , P. W. T. Pong , IEEE Trans. Magn. 2019, 55, 4, 4001809.

[4] A. S. Edelstein , G. A. Fischer , M. Pedersen , E. R. Nowak , S. F. Cheng , C. A. Nordman , J. Appl. Phys. 2006, 99, 08B317.

[5] M. E. Kiziroglou , S. W. Wright , E. M. Yeatman , Sens. Actuators, A 2020, 313, 112206.

[6] S. W. Wright , M. E. Kiziroglou , S. Spasic , N. Radosevic , E. M. Yeatman , IEEE Sensors Lett. 2019, 3, 6001104.

[7] X. Sun , L. Jiang , P. W. T. Pong , Microelectron. Eng. 2013, 111, 77.

[8] S. Yuan , Y. Huang , J. Zhou , Q. Xu , C. Song , P. Thompson , IEEE Trans. Power Electron. 2015, 30, 6191.

[9] Y. Wu , L. Xiao , S. Han , J. Chen , IEEE Transactions on Appl. Superconductivity 2025, 35, 1100205.

[10] Y. Wu , L. Xiao , S. Han , J. Chen , Cryogenics 2024, 139, 103810.

[11] F. Maspero , G. Gatani , S. Cuccurullo , R. Bertacco , in 2021 IEEE 34th Int. Conf. on Micro Electro Mechanical Systems (MEMS) , Gainesville, FL, USA 2021, pp. 374–377.

[12] Z. Marinho , S. Cardoso , R. Chaves , R. Ferreira , L. V. Melo , P. P. Freitas , J. Appl. Phys. 2011, 109, 07E521.

[13] J. Valadeiro , D. C. Leitao , S. Cardoso , P. P. Freitas , IEEE Trans. Magn. 2017, 53, 4003805.

[14] J. D. Lopez , A. Dante , A. O. Cremonezi , R. M. Bacurau , C. C. Carvalho , R. C. da Silva Barros Allil , E. C. Ferreira , M. M. Werneck , IEEE Sens. J. 2020, 20, 3572.

[15] H. Fan , S. Li , V. Nabaei , Q. Feng , H. Heidari , IEEE Sens. J. 2020, 20, 9919.

[16] L. Yang , K. Sun , M. Pan , X. Zhang , J. Peng , Y. Hu , J. Hu , W. Qiu , P. Li , IEEE Sens. J. 2023, 23, 240.

[17] M. E. Kiziroglou , Cowell M. , Kumaravel B. T. , Boyle D. E. , Evans J. W. , Wright P. K. , Yeatman E. M. , J. Phys.: Conf. Ser. 2018, 1052, 012026.

[18] A. E. Espe , T. S. Haugan , G. Mathisen , IEEE Trans. Power Electron. 2022, 37, 8659.

[19] X. Zhang , K. Youcef‐Toumi , IEEE/ASME Transactions on Mechatronics 2022, 27, 4718.

[20] M. Ali , F. Ahmad , Mater. Manuf. Processes 2019, 34, 1580.

[21] F.‐H. Wang , C.‐Y. You , N. Tian , H.‐G. Liu , J. Zhang , X.‐P. Zhu , J. Alloys Compd. 2024, 990, 174486.

[22] J. Cañada , S. F. Nagle , N. Vidal , J. M. López‐Villegas , L. F. Velásquez‐García , in 2024 IEEE 23rd Int. Conf. on Micro and Miniature Power Systems, Self‐Powered Sensors and Energy Autonomous Devices (PowerMEMS) , Tonsberg, Norway 2024, pp. 255–258.

[23] J. Cañada , K. Hyeonseok , L. F. Velásquez‐García , Virtual Phys. Prototyping 2024, 19, 2310046.

[24] S. W. Wright , M. E. Kiziroglou , E. M. Yeatman , in 2024 IEEE 23rd Int. Conf. on Micro and Miniature Power Systems, Self‐Powered Sensors and Energy Autonomous Devices (PowerMEMS) , Tonsberg, Norway 2024, pp. 243–246.

[25] S. W. Wright , M. E. Kiziroglou , E. M. Yeatman , IEEE Sens. J. 2023, 23, 20474.

[26] R. Budakian , A. Finkler , A. Eichler , M. Poggio , C. L. Degen , S. Tabatabaei , I. Lee , P. C. Hammel , S. P. Eugene , T. H. Taminiau , R. L. Walsworth , P. London , A. Bleszynski Jayich , A. Ajoy , A. Pillai , J. Wrachtrup , F. Jelezko , Y. Bae , A. J. Heinrich , C. R. Ast , P. Bertet , P. Cappellaro , C. Bonato , Y. Altmann , E. Gauger , Nanotechnology 2024, 35, 412001. 10.1088/1361-6528/ad4b23 38744268

[27] L. M. Bollig , P. J. Hilpisch , G. S. Mowry , B. B. Nelson‐Cheeseman , J. Magn. Magn. Mater. 2017, 442, 97.

[28] B. Khatri , K. Lappe , D. Noetzel , K. Pursche , T. Hanemann , Materials 2018, 11, 189. 29370112 10.3390/ma11020189 PMC5848886

[29] D. Bowen , D. Basu , IEEE Trans. Magn. 2019, 55, 8400705.

[30] Á. Díaz‐García , J. Y. Law , M. Felix , A. Guerrero , V. Franco , Mater. Des. 2022, 219, 110806.

[31] C. Ding , L. Liu , Y. Mei , K. D. T. Ngo , G. Q. Lu , in 2018 IEEE Applied Power Electronics Conf. and Exposition (APEC) , San Antonio, TX, USA, March 2018, pp. 615–618.

[32] L. Liu , T. Ge , K. D. T. Ngo , Y. Mei , G. Q. Lu , IEEE Magnetics Letters 2018, 9, 5102705.

[33] L. Liu , K. D. T. Ngo , G. Q. Lu , IEEE Trans. Magn. 2020, 56, 2000307.

[34] A. P. Taylor , C. V. Cuervo , D. P. Arnold , L. F. Velásquez‐García , J. Microelectromech. Syst. 2019, 28, 481.

[35] S. W. Wright , M. E. Kiziroglou , E. M. Yeatman , in 2022 21st Int. Conf. on Micro and Nanotechnology for Power Generation and Energy Conversion Applications (PowerMEMS) , Salt Lake City, UT, USA 2022, pp. 82–85.

[36] M. Blagojevic , A. Dieudonne , L. Kamecki , M. E. Kiziroglou , K. Krastev , D. Marty , D. Piguet , S. Spasic , S. W. Wright , E. M. Yeatman , IEEE Trans. Aerosp. Electron. Syst. 2023, 59, 3345.

[37] N. Trnka , J. Rudolph , R. Werner , in 2020 IEEE 29th Int. Symposium on Industrial Electronics (ISIE) , Delft, Netherlands 2020, pp. 326–331.

